# An Integrative Genomic and Epigenomic Approach for the Study of Transcriptional Regulation

**DOI:** 10.1371/journal.pone.0001882

**Published:** 2008-03-26

**Authors:** Maria E. Figueroa, Mark Reimers, Reid F. Thompson, Kenny Ye, Yushan Li, Rebecca R. Selzer, Jakob Fridriksson, Elisabeth Paietta, Peter Wiernik, Roland D. Green, John M. Greally, Ari Melnick

**Affiliations:** 1 Department of Developmental and Molecular Biology, Albert Einstein College of Medicine, Bronx, New York, United States of America; 2 Department of Biostatistics, Virginia Commonwealth University, Richmond, Virginia, United States of America; 3 Department of Molecular Genetics, Albert Einstein College of Medicine, Bronx, New York, United States of America; 4 Department of Epidemiology and Population Health, Albert Einstein College of Medicine, Bronx, New York, United States of America; 5 Roche NimbleGen, Inc. Madison, Wisconsin, United States of America; 6 Our Lady of Mercy Comprehensive Cancer Center, Bronx, New York, United States of America; 7 Department of Medicine, Albert Einstein College of Medicine, Bronx, New York, United States of America; Tel Aviv University, Israel

## Abstract

The molecular heterogeneity of acute leukemias and other tumors constitutes a major obstacle towards understanding disease pathogenesis and developing new targeted-therapies. Aberrant gene regulation is a hallmark of cancer and plays a central role in determining tumor phenotype. We predicted that integration of different genome-wide epigenetic regulatory marks along with gene expression levels would provide greater power in capturing biological differences between leukemia subtypes. Gene expression, cytosine methylation and histone H3 lysine 9 (H3K9) acetylation were measured using high-density oligonucleotide microarrays in primary human acute myeloid leukemia (AML) and acute lymphocytic leukemia (ALL) specimens. We found that DNA methylation and H3K9 acetylation distinguished these leukemias of distinct cell lineage, as expected, but that an integrative analysis combining the information from each platform revealed hundreds of additional differentially expressed genes that were missed by gene expression arrays alone. This integrated analysis also enhanced the detection and statistical significance of biological pathways dysregulated in AML and ALL. Integrative epigenomic studies are thus feasible using clinical samples and provide superior detection of aberrant transcriptional programming than single-platform microarray studies.

## Introduction

Regulation of gene expression involves multi-layered mechanisms in which epigenetic modifications such as DNA methylation and histone tail modifications play a major role[1], [2]. Post-translational modifications of histones at specific residues help to determine chromatin structure and therefore accessibility to gene promoters and regulatory regions. Amongst these marks, acetylation of lysine 9 on histone H3 (H3K9 acetylation) has been linked to gene activation and active transcription[3], [4]. Cytosine methylation at promoter regions, on the other hand, is associated with gene silencing[5]. Epigenetic regulation of gene expression has additional complexities; not only is the presence of specific epigenetic marks important but their localization and density also seem to play a crucial role[6]–[8].

Disruption of epigenetic regulation during malignant transformation can profoundly alter a cellular phenotype, resulting in aberrant cellular proliferation and survival. Epigenetic dysregulation is currently recognized as one of the hallmarks of cancer [9], [10]. DNA methylation at promoter regions of key negative cell cycle regulators and DNA repair genes leads to their abnormal epigenetic silencing in many neoplasms[5], [11]–[14]. However, it is not clear whether this aberrant DNA methylation pattern is sufficient to determine gene silencing, or whether it is in fact part of a more complex process involving chromatin remodeling factors and changes in histone modifications[15], [16].

Gene expression profiling studies have been performed with the aim of dissecting the molecular subtypes of several neoplasms, in an effort to predict accurately tumor behavior and to identify important oncogenic genes and biological pathways. These studies have revealed the presence of unique gene expression signatures distinguishing specific subgroups of cancers and have served to improve our understanding of the biology of these diseases (e.g. [17]–[20]). However, only part of the cellular information is contained at the messenger RNA level, and transcriptional activity is dependent on multiple factors. Among these factors are epigenetic marks, such as cytosine methylation and histone tail modifications, which help to determine and regulate chromatin structure and function including gene expression.

Therefore, while gene expression studies using DNA microarrays have had a great impact in the study of cancer, it is important to recognize that there are limitations associated with this technique. Firstly, gene expression microarrays capture a snapshot of the cell's transcriptome, detecting genes being actively transcribed at the time of RNA extraction, but they do not capture any information concerning the genes' regulatory states and consequently their potential for transcriptional changes in response to stimuli. For example, a locus such as the *O* ^6^-methylguanine DNA methyltransferase *(MGMT)* gene is not prognostically useful in terms of its basal expression state [21] but the cytosine methylation status of its promoter provides an excellent indicator of how well gliomas will respond when treated by alkylating agents [22]. We hypothesize that biologically significant changes in expression can be missed by expression arrays due to technical limitations, but might be captured by epigenomic studies by identifying genes at which promoter cytosine methylation or H3K9 acetylation differ and testing them with highly-quantitative techniques.

In order to test these hypotheses, we carried out genome-wide studies for DNA methylation and H3K9 acetylation as well as gene expression microarrays in patients with acute myeloid and lymphoblastic leukemia (AML and ALL, respectively). These cell types were chosen so that we could test whether the technical approach we were exploring was feasible in typical clinical samples, using cell types that should be markedly distinctive. We show here that the integration of the information captured by these different platforms results in a more comprehensive detection of differentially regulated genes and an enhancement of the apparent biological relevance of the findings.

## Results

### Multiplatform epigenomic microarray analysis can be performed on routine leukemia clinical samples

Bone marrow aspirates from three adult patients with AML and two patients with ALL were enriched for mononuclear cells by Ficoll gradient separation to yield >90% leukemia blast cells (**see** **Table 1** **for patient characteristics**). Frozen aliquots of these samples were thawed for analysis using three different microarray platforms. Unsheared high quality genomic DNA was extracted from 5–10 million cells for genome-wide cytosine methylation analysis using the HELP (**H**paII tiny fragment **e**nrichment by **l**igation mediated **P**CR) assay[23]. Ten million cells were cross-linked by exposure to formaldehyde for ChIP-chip with a specific antibody for histone H3 lysine 9 (H3K9) acetylation, a specific antibody for total histone H3 and non-specific rabbit IgG. Total RNA was extracted from 20 million cells using Qiagen's RNeasy mini kit for expression array analysis.

**Table 1:** Patients' characteristics

	Patient 1	Patient 2	Patient 3	Patient 4	Patient 5
**Age (yrs)**	54	82	25	44	53
**Gender**	Female	Female	Male	Female	Female
**Diagnosis**	ALL	ALL	AML	AML	AML
**Karyotype**	t(9;22)(q34;q11)	t(9;22)(q34;q11.2), add(16)(q21), −20, +mar	46,XY, t(5;21;8)(q15;q22;q22)	NA	t(10;11)(q22;q23)
**Immunophenotype**	Early Pre-B ALL with co-expression of CD33 and CD13	Early Pre-B ALL with co-expression of CD33 and CD13	Undifferentiated AML CD65 negative, CD19 positive, CD11a negative	CD11b Positive, co-expression of CD7	Undifferentiated AML, CD65 negative
**Molecular characterization**	BCR-ABL p190 (e1a2)	BCR-ABL p190 (e1a2)	AML1/ETO positive	negative by RT-PCR for BCR/ABL, AML1/ETO, CBFbeta/MYH11, MLL-TD and FLT3-ITD	negative by RT-PCR for BCR/ABL, AML1/ETO, CBFbeta/MYH11, MLL-TD and FLT3-ITD

The HELP cytosine methylation analysis was performed in triplicate for each sample. Methylation status could be assigned in >86% of probe sets (i.e. 1–14% of probe sets failed to provide a clear signal). The correlation between cytosine methylation profiles in individual replicates from the same sample was r = 0.93–0.99 and correlation amongst different patients was r = 0.78–0.91 (**Figure S1A**). After initial quality control one replicate from ALL-2 was excluded from further analysis. ChIP-chip was performed in duplicates and the correlation between replicates was r = 0.92–0.93, while correlation between different patients ranged between r = 0.88–0.92 (**Figure S1B**). After initial quality control one replicate from ALL-2 and one from AML-3 were excluded from further analysis. Gene expression analysis was performed in quadruplicate and the best two replicates for each patient were selected based on our initial quality control (see methods section). Correlation between replicates was r = 0.95–0.99. Correlation between the five patients in gene expression ranged from r = 0.84–0.96. (**Figure S1C**). For each platform, single locus gene validation was performed on a subset of genes respectively by MassArray for methylation (9 genes), by qChIP for H3K9 acetylation (10 genes), or qRT-PCR for gene expression (15 genes), demonstrating that each microarray platform was highly accurate in predicting the actual abundance of mRNA, DNA methylation and histone acetylation of each gene (**Figures S2A, S2B and S2C**). Taken together, these data indicate that multi-array analysis is robust and feasible in frozen leukemia patient samples.

### ALL and AML have distinct DNA methylation profiles

**Figure 1:**
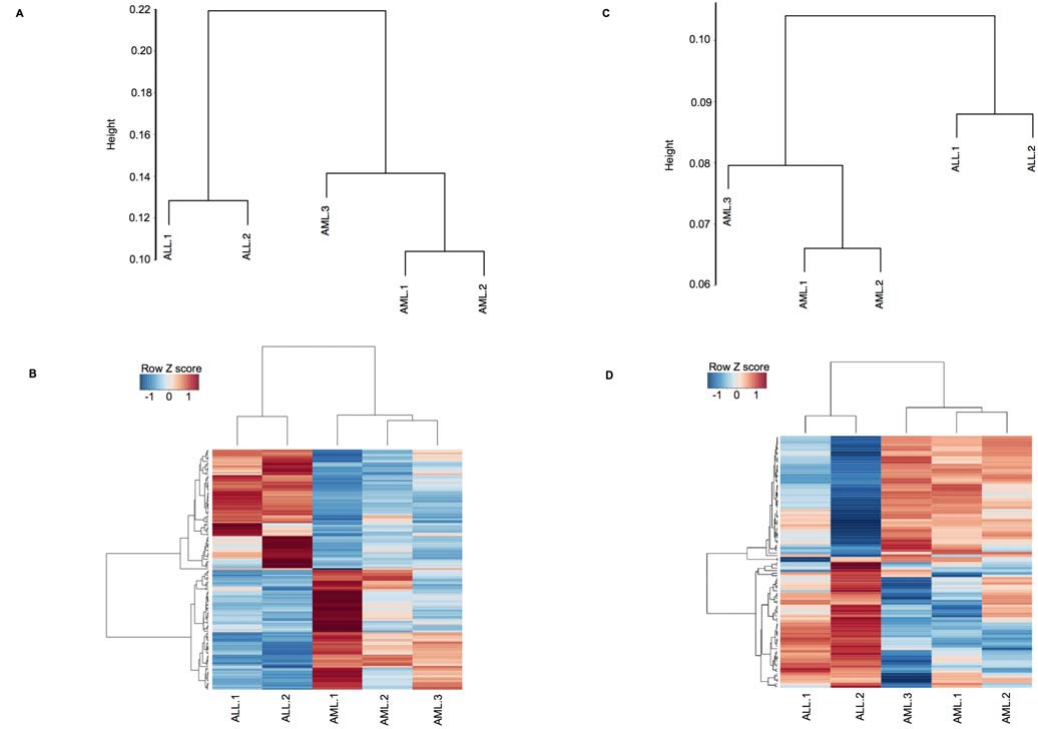
Epigenomic platforms readily classify leukemia samples according to lineage. Unsupervised clustering of DNA methylation by HELP and H3K9 acetylation ChIP-chip data succeeded in accurately segregating the samples according to their lineage. Panel A: Dendrogram representing the result of hierarchical clustering of leukemia samples using DNA methylation data. The scale on the left represents the correlation distance metric. Panel B: Heatmap of top 150 genes from the first principal component of correspondence analysis, which separated ALL samples from AML. Genes are shown on the rows and samples on the columns, and data were row-centered. Low values corresponding to greater methylation are represented in blue and high values corresponding to less methylation are in red. Panel C: Hierarchical clustering of leukemia samples using H3K9 acetylation ChIP-chip. Panel D: Heatmap of top 100 genes from the first principal component of correspondence analysis, which separated ALL samples from AML. Low values corresponding to less H3K9 acetylation are represented in blue and high values corresponding to greater H3K9 acetylation are in red.

Although aberrant DNA methylation is known to occur in human leukemias,[24] it is not known whether (as has been shown for gene expression profiling) different types of leukemia have specific and distinct profiles of promoter DNA methylation. All five leukemia samples presented a bimodal distribution of DNA methylation, with approximately two-thirds of HpaII fragments detected as methylated and one-third as hypomethylated. In order to determine whether AML and ALL samples display distinct DNA methylation signatures, we performed unsupervised clustering of HELP array data. The DNA methylation profiles from leukemia samples were submitted to three different unsupervised clustering algorithms including hierarchical clustering (HC), principal component analysis (PCA) and correspondence analysis (COA). Each of these methods detects global differences between samples as distances and represents them in different ways. HC of HELP profiles separated the cases into two separate nodes containing either the ALLs or the AMLs. (**Figure 1A**). Similar to HC, PCA yielded a clear difference between AML and ALL (**Figure S3A**). COA revealed an identical node structure as HC and allowed us to generate a list of genes differentially methylated between AML and ALL (**Figure 1B**). All three of these clustering methods demonstrated that promoter methylation signatures could accurately segregate the samples into the AML and ALL categories. Since there are 10 distinct ways in which 5 samples can be divided into two groups of 2 and 3 samples each, it was calculated that there was a 1 in 10 chance that this separation of the samples by lineage was due to chance.

### Histone 3 lysine 9 acetylation profiles distinguish ALL from AML cell types

In contrast to DNA methylation, acetylation of histone H3 on lysine 9 has been associated with genes that are transcriptionally active[3], [4]. We predicted that the epigenetic information contained in the H3K9 acetylation status of genes would be informative regarding the biological phenotype of AML and ALL. H3K9 acetyl ChIP-chip performed robustly using patient samples and revealed a bimodal distribution in which approximately one-third of all gene promoters represented displayed H3K9 acetylation. In order to determine whether H3K9 acetylation status could distinguish AML from ALL cells, unsupervised clustering (HC, PCA and COA) was again performed (**Figures 1C & 1D** **and Figure S3B**). All three methods yielded a similar segregation of the cases into two classes consistent with their biological lineages. Taken together, these data indicate that AML and ALL are epigenetically distinct cell types that can be classified through genome-wide analysis of DNA methylation and H3K9 acetylation.

### DNA methylation, histone acetylation and gene expression profiling identify distinct cohorts of genes in AML and ALL cells

**Figure 2:**
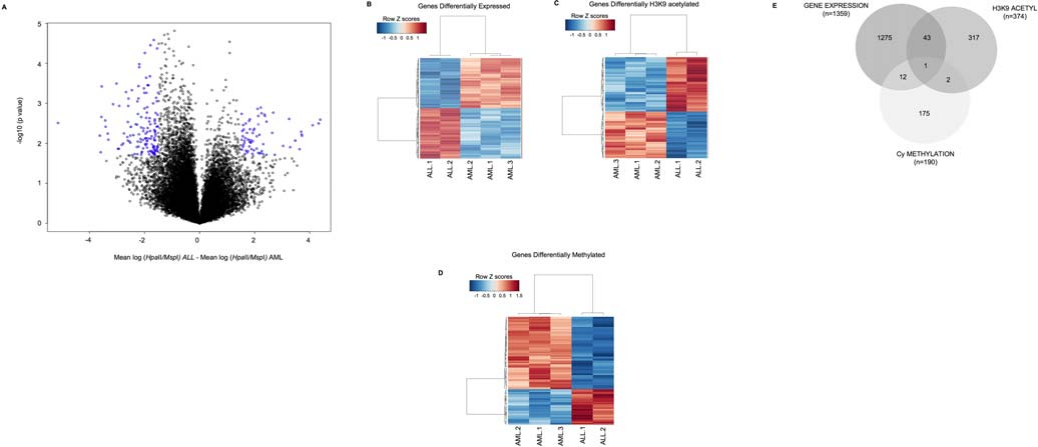
Gene expression, DNA methylation and H3K9 acetylation identify partially overlapping genes signatures. Supervised analysis of the three datasets using a T-test and leukemic lineage as the dichotomous variable identified partially overlapping gene signatures distinguishing between ALL and AML. Panel A: Plot of differences between sample means (x axis) vs. −log_10_ p values (y axis), illustrating the two criteria required for significance in HELP data. Blue dots represent genes with p<0.02 and a difference in sample means>1.5. Panel B: Heatmap of gene signature identified using gene expression arrays. Genes are shown on the rows and samples on the columns, and data were row-centered. Low values of gene expression are represented in blue and high values are in red. Panel C: Heatmap of gene signature identified using H3K9 acetylation arrays, constructed as described for Panel A. Low values corresponding to less H3K9 acetylation are represented in blue and high values corresponding to greater H3K9 acetylation are in red. Panel D: Heatmap of gene signature identified using DNA methylation (HELP) arrays, constructed as described for Panel A. Low values corresponding to greater methylation are represented in blue and high values corresponding to less methylation are in red. Panel E: Venn diagram illustrating overlap between the three gene signatures.

We next asked whether genes detected as robustly differentially methylated, H3K9 acetylated and expressed by microarray analysis would be overlapping or complementary. In order to identify the genes that most significantly distinguish AML from ALL cells in terms of gene expression, H3K9 acetylation and DNA methylation, we performed a supervised analysis using a T-test, with lineage as the dichotomous variable. For DNA methylation data, a second condition requiring a minimum difference of 1.5 between the two sample means was implemented in order to maximize the capture of biologically significant changes in methylation (**Figure 2A**). Analysis was restricted to the 17,180 genes that are represented on all three microarrays platforms used. Using this common denominator, 1,359 genes were identified as differentially expressed between the two types of leukemia at a significance level of p<0.02. Histone H3 lysine 9 acetylation, on the other hand, identified 374 genes at the same level of significance, while 190 genes were classified as differentially methylated (**Figures 2B, 2C and 2D**). Overlap between gene expression and H3K9 acetylation signatures included 45 genes, overlap between DNA methylation and gene expression included 13 genes, while DNA methylation and H3K9 acetylation overlapped on 3 genes. All three platforms overlapped on only 1 gene: the metallopeptidase *LMLN*. These partially overlapping signals probably result from both the distinct biological nature of the parameters being measured by each different platform as well as from unique technical limitations that affect each one. Thus, gene signatures obtained using epigenomic and gene expression profiling can provide complementary sets of genes (**Figure 2E**), resulting in a greater set of unique biological features being captured, which would otherwise be missed by any single platform.

### H3K9 acetylation widely correlates with gene expression

**Figure 3:**
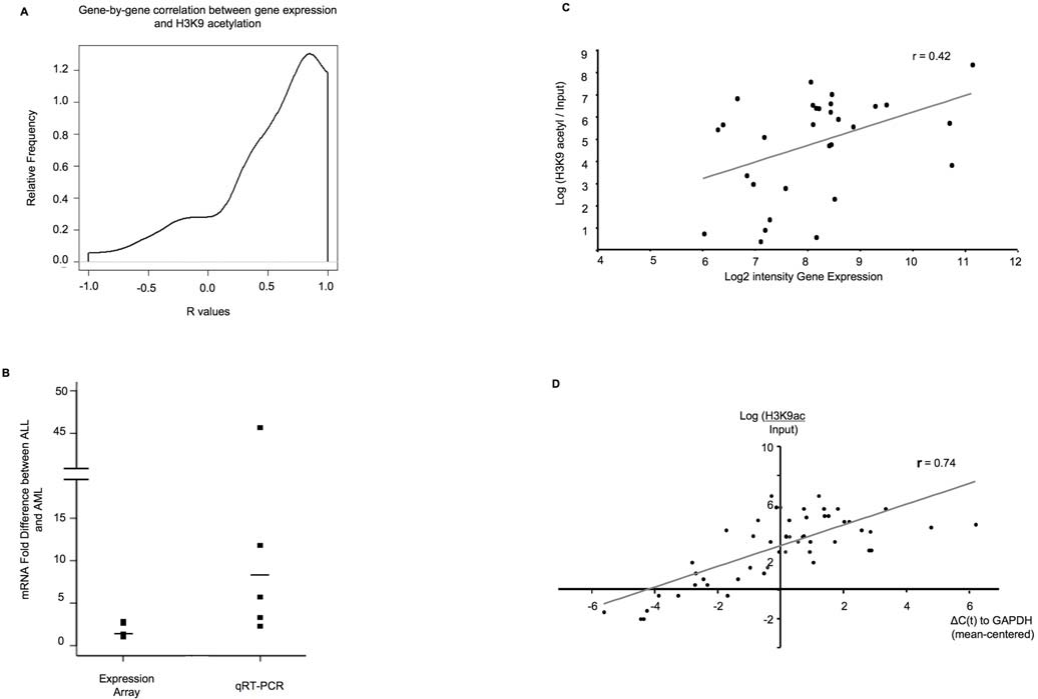
H3K9 acetylation correlates with active gene expression and can help rescue genes from within the noise level of gene expression arrays. Panel A: Smoothed histogram of gene-by-gene correlation between gene expression and H3K9acetyl ChIP-chip for genes displaying a high SNR on both platforms. Median correlation was r = 0.84. Panel B: Comparative fold difference in mRNA levels (y axis) as detected by gene expression arrays and qRT-PCR for five genes that displayed differential H3K9 acetylation but had not been detected as differentially expressed on the microarrays. Panels C and D: Correlation for those same 5 genes between H3K9 acetylation levels (y axis) and gene expression levels (x axis) measured by either gene expression arrays (panel C) or qRT-PCR (panel D). Correlation between H3K9 acetylation and gene expression was significantly higher when the latter was measured by qRT-PCR, indicating that H3K9 acetyl ChIP-chip could predict genuine differences in gene expression levels that were missed by gene expression arrays, probably due to relative compression of the signal on the gene expression arrays as demonstrated in panel B.

Acetylation of lysine 9 on histone H3 has been associated with actively transcribed genes [3], [4], but the degree of association is unknown at the genome-wide level in leukemia patients. In order to define with greater precision the quantitative relation between variation in histone acetylation and gene expression, we studied the correlation between promoter H3K9 acetylation and gene transcript abundance. Since correlations between variables are degraded rapidly by increasing noise levels, we selected genes with a SNR>2.5. The median correlation between H3K9 acetylation and gene expression over 393 genes with a SNR>2.5 in both measures was 0.86 (**Figure 3A**). In order to test whether these findings could be simply due to an artifact of the small sample size, we developed a statistical model in which we selected genes that were as much as possible statistically independent and then re-calculated the correlations after several sample-label permutations. This analysis showed that the probability of obtaining the observed correlations by chance was astronomically small (p<1 in 108) **(see supplementary methods).** Therefore, the degrees of H3K9 acetylation and gene transcript level are quantitatively tightly linked in these leukemic cells.

Since microarray measures are inherently corrupted by noise, we hypothesized that the true underlying biological correlations between gene expression and H3K9 acetylation were much greater than captured through a simple correlation of high SNR genes. In other words, that this epigenetic modification could detect differences in gene expression missed by expression arrays. In order to test this hypothesis, we randomly selected a group of five genes (*FER, HOXA1, DOCK5, LT4R1* and *PAX5*) among those that presented differential H3K9 acetylation with an SNR>2.5, but that were not detected as differentially expressed by microarray analysis. A more precise measurement of transcript abundance of these genes was performed by qRT-PCR, which revealed that differential expression was significantly greater than had been detected by microarray: the range of fold difference between ALL and AML by gene expression array was 1.0–2.8 and by qRT-PCR 2.3–45.6 (**Figure 3B**). Furthermore, the correlation between H3K9 acetylation and gene expression measured by qRT-PCR was considerably higher than between H3K9 acetylation and array gene expression (r = 0.74 vs. 0.42, respectively) (**Figures 3C and 3D**). The data suggest that significant differential gene expression can be missed by expression microarray analysis but recaptured by integrating functionally relevant epigenetic modifications such as H3K9 acetylation. These data also confirm the close association between transcriptional activation and histone acetylation at the genome-wide level.

### Genome-wide promoter methylation status shows an overall inverse correlation with gene expression and H3K9 levels

**Figure 4:**
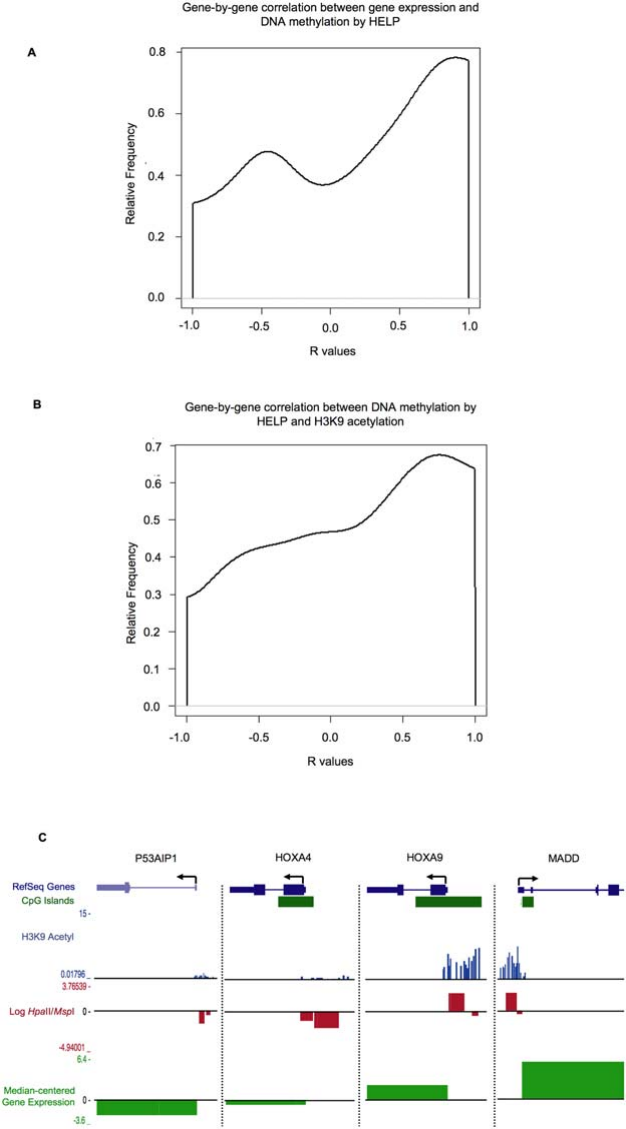
Promoter DNA methylation shows genome-wide inverse correlation with gene expression and H3K9 acetylation: Panel A: Smoothed histogram of gene-by-gene correlations between log(HpaII/MspI) values and gene expression, showing a positive correlation between the two measures for the majority of genes, which translates into a negative biological correlation (*i.e.* higher promoter methylation correlates with lower gene expression). Panel B: Smoothed histogram of gene-by-gene correlations between log(HpaII/MspI) values and H3K9 acetylation, showing a positive correlation between the two measures for many of the genes, which translates into a negative biological correlation (*i.e.* higher promoter methylation correlates with lower promoter H3K9 acetylation). Panel C: Graphical representation of the data from all three platforms for one of the cases (AML.2) as custom tracks in the UCSC genome browser[40]. Four representative genes are shown here to illustrate the correlation between the three platforms. H3K9 acetylation data (in blue) is represented as the ratio of the signal between the H3K9 acetyl channel and the input channel; DNA methylation (in red) is represented as log(HpaII/MspI), so that a negative deflection corresponds to a methylated HpaII fragment while a positive one corresponds to a hypomethylated fragment; finally, gene expression data (in green) is represented as median-centered log_2_ of RMA-normalized intensities.

Promoter DNA methylation is generally believed to be associated with gene silencing, although it is not clear whether this can be generalized in a whole genome analysis. Therefore, we determined the relationship between gene expression and DNA methylation in our set of leukemia patient samples. As before, high SNR genes (>2.5) were selected for this analysis. Correlation of expression and DNA methylation among these genes revealed a bimodal distribution where two-thirds of genes displayed a strong positive correlation between expression and the log HpaII/MspI ratio (which translates biologically into an inverse correlation between gene expression and DNA methylation levels) where the peak value was r = 1.0. The remaining one-third of genes showed a weak negative correlation (peak at r = −0.5) (**Figure 4A**). The presence of a strong correlation between gene expression and log HpaII/MspI ratio was still clearly detected even at SNR cutoffs of 1.3. These data indicate that for a majority of promoters, DNA methylation is strongly associated with gene silencing. A similar bimodal distribution was detected for the correlations between DNA methylation and H3K9 acetylation. The largest peak of the correlations was found at r = 1.0. A second and less defined peak that represented a smaller population of genes was found with a slightly negative correlation (around r = −0.5) (**Figure 4B**). While the first peak is to be expected, representing silenced genes with methylated promoters lacking the H3K9ac mark associated with active chromatin, the second peak may be explained by a number of different factors (see discussion). Overall, the results demonstrate that for most genes, the presence of high levels of methylation corresponds with repression, while high levels of acetylation correspond to activation, as exemplified in **Figure 4C**.

### Integration of histone acetylation and gene expression profiling synergistically enhances the detection of differentially regulated genes between AML and ALL samples

**Figure 5:**
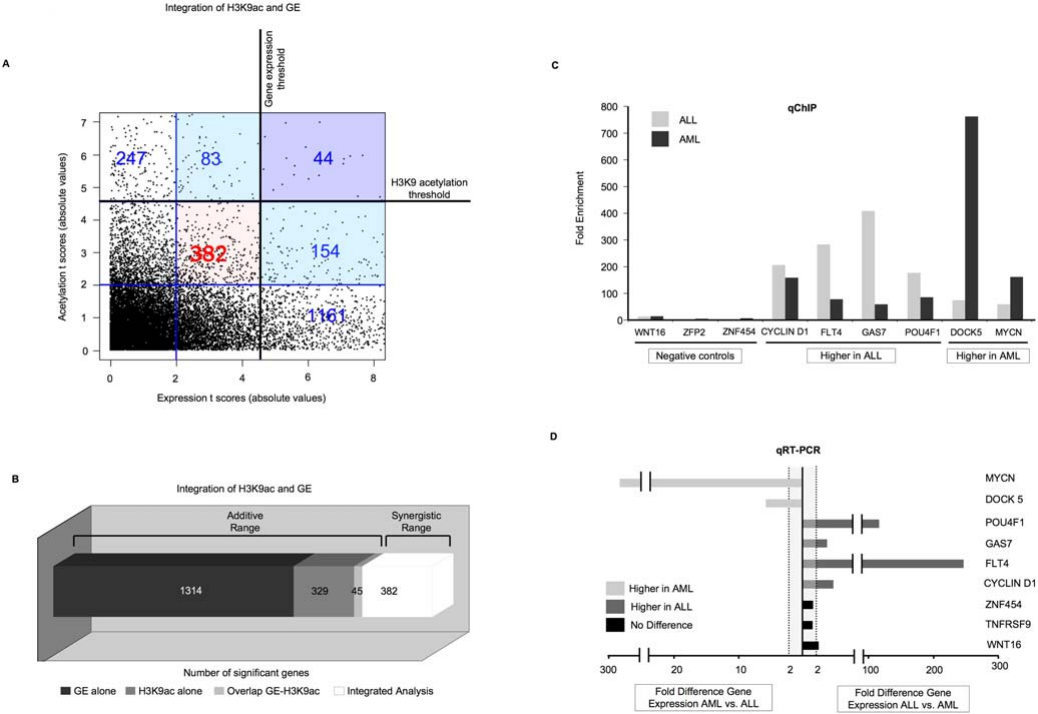
Gene expression and H3K9 acetylation synergize to increase the number of differentially expressed genes identified. Panel A: Dot plot of t scores from T-test for gene expression (x axis) and H3K9 acetylation (y axis). 1,359 genes were identified as differentially expressed and 374 as differentially H3K9 acetylated, with absolute t scores>4.5 (p<0.02). 44 genes crossed the threshold for both assays (upper right). 83 genes that passed H3K9 acetylation threshold displayed marginal t scores (>2 and <4.5) for expression, while 154 genes that passed expression threshold displayed marginal t scores (>2 and <4.5) for H3K9 acetylation. Differential expression of the former genes was validated as in Fig 4. Combining genes with marginal t scores (>2 and <4.5) for both platforms simultaneously yielded an additional 382 genes with positive correlation that would have been missed by both platforms (central square, red). For illustrating purposes, points corresponding to gene expression t scores>8 or H3K9 acetylation t scores>7 were excluded from the figure. Panel B: Bar graph illustrating synergism between the gene expression and H3K9 acetylation analyses. 1314 genes were identified only by gene expression, 329 by H3K9 acetylation alone, and 44 genes were captured by both platforms. After the integrated analysis, an additional 382 genes were identified. Panels C and D: Validation of fold enrichment for H3K9 acetylation by qChIP (panel C) and fold difference in expression by qRT-PCR (panel D) of 9 genes (6 test and 3 negative controls) identified by the integrated analysis that had been previously missed by both.

Taken together with the fact that H3K9 acetylation and gene expression capture different cohorts of genes, the above findings led us to predict that integrating the measurement of individual gene variation captured by H3K9 acetylation and gene expression profiles would recover biologically significant differences missed by both platforms when considered independently of each other. In order to determine if this was the case, we tested whether integration of gene expression and H3K9 acetylation could enhance the discovery of genes that discriminate between AML and ALL cells. As mentioned above, a T-test analysis (p<0.02 (absolute t score>4.5)) identified 1,359 differentially expressed genes and 374 differentially acetylated, with an overlap of 45 genes (**Figure 2D**). We next looked for genes that did not meet these criteria for significance on either platform, but that were marginally significant for both of them (p>0.02 and <0.14; absolute t score<4.5 and >2 with both t scores displaying the same direction). Applying these criteria allowed identification of an additional 382 genes that are likely to be discriminative between ALL and AML (**Figures 5A and 5B**). Since the noise in each platform is expected to be random, genes that in both platforms manage to reach a borderline level of significance are unlikely to be due to random chance. Based on the integration of their individual t-scores, the probability that such genes are differentially regulated is increased to p<0.01 (p<(0.14^2^)/2). Nine genes (6 test and 3 negative controls) were chosen for validation by single locus quantitative ChIP (qChIP) and qRT-PCR. All six test genes were found to be both differentially H3K9 acetylated (**Figure 5C**) and differentially expressed (**Figure 5D**), while none of the negative controls were found to show any significant difference by either qChIP or qRT-PCR. Due to the limiting amount of sample available we were unable to perform replicate PCR runs, but each qPCR experiment was itself ran in triplicate. The data suggest that integrated analysis captures biological differences between tumors otherwise missed by independent analysis of gene expression or histone acetylation.

### Integration of DNA methylation and gene expression profiling has an additive effect on the detection of differentially regulated genes between AML and ALL samples

**Figure 6:**
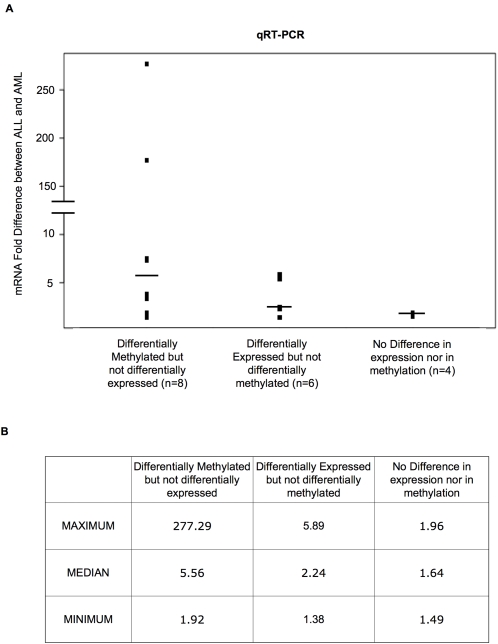
DNA methylation identifies differentially expressed genes missed by gene expression arrays. Panel A: Fold difference in gene expression by qRT-PCR between ALL and AML in a subset of genes identified as differentially methylated but not as differentially expressed (left), a group of genes that had been identified as differentially expressed but not differentially methylated (center), and in a group of genes that had not been identified as either differentially expressed nor differentially methylated. Panel B: Table showing the median, maximum and minimum values in fold difference for the three groups, reflecting how a subgroup of genes that is missed by gene expression arrays but identified by DNA methylation display comparable differences in gene expression levels when using a more sensitive technique such as qRT-PCR.

We next wished to determine if DNA methylation profiles, like H3K9 acetylation, could enhance the capture of differentially-regulated genes. The same analytical approach to the integration of gene expression and DNA methylation was used to quantify to what extent these two platforms reinforced each other in the discrimination of leukemic lineage of our samples. An initial significance level of p<0.02 (absolute t score>4.5) was once again chosen, but an additional requirement of a difference between the two means greater than 1.5 was applied to the methylation data as described above. In this way, 1,359 genes were differentially expressed and 190 were differentially methylated between ALL and AML cells with an overlap of 13 genes. In order to test whether the two platforms reinforced each other, we examined how many genes were consistently marginally significant on both platforms (p>0.02 and <0.14; absolute t score<4.5 and >2). However, in order to ensure a potentially biologically relevant difference in methylation, we once more had an additional criterion of a minimum difference between the sample means>1.5 for the methylation data. When these criteria were applied to the integration of gene expression and DNA methylation we did not find a significant number of additional genes. However, when qRT-PCR was performed on a subset of genes (eight genes) that were identified as differentially methylated but that were not predicted as differentially expressed on the arrays, significant actual differences in mRNA levels between ALL and AML cells were found for all of these genes. This level of differential gene expression by qRT-PCR was comparable to that measured for genes that had been successfully identified as differentially expressed by the expression arrays (**Figure 6A and 6B**). These results suggest that the HELP platform is capable of rescuing differentially expressed genes. However, it does so in an additive fashion with gene expression arrays as opposed to the synergistic results obtained with the integration of gene expression and H3K9 acetylation.

### Integration of platforms improves identification of biological pathways that are differentially regulated in AML versus ALL cells

Our next goal was to determine whether over-representation of particular gene networks and canonical pathways could be found among the genes identified by the different platforms. For this purpose we used the Ingenuity Pathway Analysis software (Redwood City, CA). We compared and contrasted the biological gene networks captured as being differentially regulated in AML versus ALL cells by the three different platforms compared with those captured by integrated analysis. We found that the top networks captured by each of the three individual platforms yielded smaller and less directly affected networks centered in part around HoxA9 and APP in H3K9 acetylation, TNF and NFkB in cytosine methylation, and TERT and NFkB in gene expression (**Figure S4A**). In contrast, the integrated analysis captured gene networks which were clearly centered around TNF and TP53, including multiple directly involved genes associated with the central node (**Figure S4B**). In addition, networks of likely pathological importance (such as the one centering around MYC) were completely missed by the single platforms but were rescued when the data for the three platforms were integrated (**Figure S4C**). This result demonstrates that the integration of data from gene expression and epigenetic platforms not only rescues genes that are missed by either platform alone, but may also enhance their power to more accurately define the most highly affected biological pathways.

**Figure 7:**
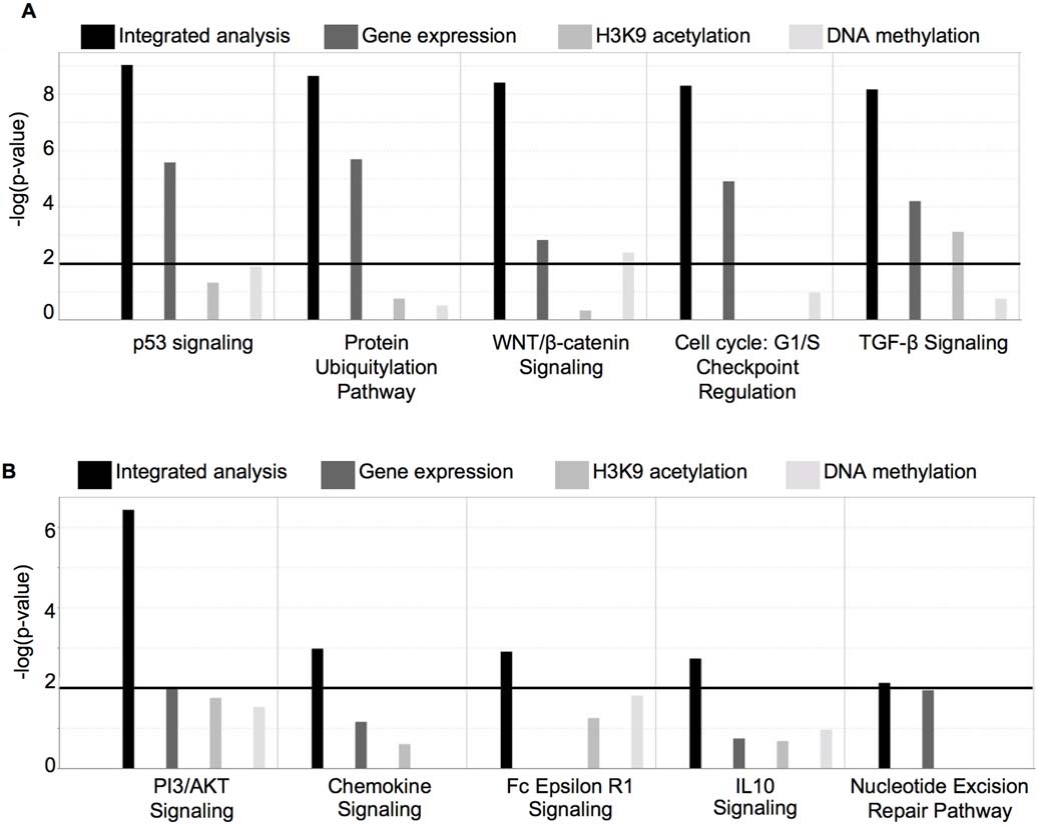
Integration of data from all three platforms enhances the biological information captured. Panel A: Bar graph comparing the significance level of enrichment (y axis: −log(p-value)) for genes within specific canonical pathways for the integrated analysis and the individual platforms. Panel B: Bar graph showing a subset of canonical pathways that were detected as significant by the integrated analysis, but that had been missed by the individual platforms.

Likewise, the integrated analysis provided a more statistically significant capture of canonical pathways (*e.g.* p = 9.5×e^−10^ for the TP53 signaling pathway, p = 2.33×e^−9^ for the protein ubiquitylation pathway and p = 4.03×e^−9^ for the WNT-β catenin signaling pathway) than any single platform (**Figure 7A** **and** **Table 2**), and rescued to the analysis another group of pathways that would otherwise have been missed (*e.g.* the PI3K/AKT signaling pathway (p = 3.27×e^−7^) and the chemokine signaling pathway (p = 1.06×e^−3^)) (**Figure 7B**, **Table 3** **and Table S1**). These data suggest that integrative analysis provides a superior platform for hypothesis-generating experiments and potentially greater accuracy in detecting the most relevant biological processes that distinguish different tumors from each other.

**Table 2:** Enrichment of specific canonical pathways by the integrated analysis and the individual platforms

Pathway	Integrated analysis (p value)	Gene expression (p value)	H3K9 acetylation (p value)	DNA methylation (p value)
TP53 signaling	9.52×e^−10^	2.7×e^−6^	4.9×e^−2^	1.3×e^−2^
Protein ubiquitylation	2.33×e^−9^	2.08×e^−6^	1.8×e^−1^	3.1×e^−1^
WNT-β catenin signaling	4.03×e^−9^	1.5×e^−3^	4.8×e^−1^	4.2×e^−3^
G1/S checkpoint regulation	5.8×e^−9^	1.2×e^−5^	–	1.1×e^−1^
TGFβ signaling	6.99×e^−9^	6.3×e^−3^	7.77×e^−4^	1.8×e^−1^

**Table 3:** Canonical pathways rescued by the integrated analysis

Pathway	Integrated analysis (p value)
PI3K/AKT signaling	3.27×e^−7^
Chemokine signaling	1.06×e^−3^
Fc signaling	1.26×e^−3^
IL10 signaling	1.83×e^−3^
Nucleotide acid excision repair	7.4×e^−3^

## Discussion

Histone modifications and DNA methylation play a critical role in regulating gene expression by modifying the chromatin structure of genes and recruiting additional regulatory factors. Our work is based on the hypothesis that a truly integrated epigenomic analysis could yield superior insight into the transcriptional programming of cancer cells beyond that obtained by simply measuring abundance of mRNAs through expression microarrays, since epigenetic modifications will capture information not only from genes being actively transcribed, but they will also reflect the *availability* for transcription by informing on the chromatin structure at specific loci. As proof of principle, we selected two functionally validated epigenetic marks–cytosine methylation and histone 3 lysine 9 acetylation–in addition to standard gene expression arrays and tested their ability to identify gene regulatory differences between the AML and ALL cell types. First, we demonstrate that such multiplatform epigenomic studies can be readily performed in enriched leukemia cells from standard clinical trial patient specimens. Second, using a novel approach for integrated analysis, we demonstrate that not only is there a functional relationship between gene expression and epigenetic marks but, more importantly, that these platforms synergize to provide a more complete and comprehensive analysis of transcriptional programming in human cells. Some of the strengths of this study include the rigorous quality control steps, the use of a powerful DNA methylation platform on specially designed high density oligonucleotide microarrays, the use of primary patient materials, the performance of the three different assays using the same type of 50-mer high density oligonucleotide arrays in multiple replicates and the extensive single locus validation.

Recently DNA methylation microarrays have been used to study acute leukemias as well as other malignancies. Groups of hypermethylated genes have been identified by such studies in AML cell lines [25] as well as in ALL patient samples [26]. Thus, the integration of genetic and epigenetic platforms seems only natural, since individually both types of platforms have proven to capture biologically relevant information. Along these lines, some groups have begun to investigate the potential to be found in the combination of information from different microarray platforms. Shi et. al. used a CpG island microarray containing 1507 expressed CpG island sequence tags to carry out a triple analysis of histone acetylation, DNA methylation and gene expression in an ovarian cancer cell line treated with trichostatin A and 5′-deoxyazacytidine. While they were able to detect a functional interaction between histone acetylation and DNA methylation, they could not demonstrate an overall correlation between changes in epigenetic modifications and changes in expression levels [27]. Wu et. al. used a combination of ChIP-chip for H3K9 modifications and Differential Methylation Hybridization (DMH) on a 9.2 K mouse promoter array and showed an inverse correlation between H3K9 acetylation and DNA methylation, while no significant correlation could be found between DNA methylation and H3 dimethyl-K9 at the promoter level[28]. In our study, however, we propose the use of a combination of three different high-density genomic and epigenomic platforms for the in-depth analysis of their relationship in the context of human cancer specimens.

Posing a simple biological question–that is the differentiation between cell types in a sample set–we determined first by carrying out a systematic unsupervised clustering analysis that epigenomic platforms can be readily used for profiling and classification of leukemia clinical samples. Moreover, the combination of DNA methylation and H3K9 acetylation to gene expression data resulted in a significantly larger number of genes being identified that distinguished ALL from AML samples. Since each one of these platforms is affected by unique technical limitations, it is not surprising that they would result in the detection of only partially overlapping cohorts of genes. The existence of such limitations was confirmed by the fact that restricting the analysis to the subset of genes that displayed high signal to noise ratios on any two platforms (i.e. those genes that we can be certain were accurately measured by both platforms) resulted in a high degree of correlation between the different measures.

Furthermore, we hypothesized that this technical limitation due to the presence of noise in gene expression arrays was significantly affecting our ability to detect genuine differences in mRNA levels. By looking at a group of genes that displayed a significant difference between ALL and AML in either H3K9 acetylation or DNA methylation levels but did not display significant differences on gene expression arrays we found that when the mRNA levels of these genes were measured by qRT-PCR, an underlying difference in gene expression could be readily detected. Thus, we were able to confirm that there is an important degree of loss of information when carrying out genome-wide studies by gene expression microarrays alone, and that this information can be recovered by the integration of epigenetic data, reflecting the additive benefit obtained from such an integrated approach.

However, our main goal was to investigate whether gene expression and epigenomic microarrays were capable of reinforcing each other. Our data show that it is possible to further harness the power of integrated epigenomics to identify differentially regulated genes, since genes missed by both gene expression and epigenetic profiling could be recovered for analysis by taking advantage of the tendency of gene expression profiling to correlate positively or negatively with epigenetic marks. For this we looked for genes that were marginally below the significance threshold on gene expression *and* H3K9 acetylation and for which both measures behaved *concordantly*. Using these new criteria, an additional 382 genes were identified that had been missed by both platforms. Careful single locus validation of randomly selected genes from this cohort confirmed such genes to be genuinely differentially acetylated and expressed, thus demonstrating the *synergistic* power of this integrative analysis.

We propose that the additive and/or synergistic ability of integrative genomics and epigenomics to capture differentially-regulated genes in human clinical samples will enhance understanding of disease pathogenesis when carried out in an adequately designed study. The current study used the extreme comparison of ALL with AML clinical samples to demonstrate proof of principle of the approach. However, even with limited numbers of samples, the integrated analysis captures gene networks missed by single platforms, improves the level of confidence in gene networks which were only partially recognized by single platforms, and may center networks more completely around critical mediators of tumorigenesis so that subsequent functional studies could focus on gene products most likely to occupy central roles in the biology of the specific tumors.

In summary, our simple approach for integrated analysis shows a functional relationship between gene expression and epigenetic marks and, more importantly, demonstrates that these platforms synergize to provide a more complete and comprehensive analysis of transcriptional programming. We predict that when applied to large cohorts of patients enrolled in clinical trials, this integrated epigenomics approach will provide more accurate disease classification and more powerful prognostic information, which could be then used to design improved risk adapted and targeted therapy clinical trials.

## Materials and Methods

### Leukemia samples

Specimens were obtained from 5 patients diagnosed with ALL (2 cases) or AML (3 cases). All patients had signed informed consent. Use of the ALL samples was approved through the Institutional Review Board (IRB) of Our Lady Of Mercy Cancer Center and use of the AML samples was approved through the Eastern Cooperative Oncology Group IRB for the E1900 clinical trial. Furthermore, the study was also approved by the Albert Einstein College of Medicine's Institutional Review Board. Mononuclear cells were isolated from heparinized bone marrow or peripheral blood specimens by ficoll-hypaque density centrifugation. The cells were washed twice in phosphate-buffered saline and subsequently frozen at a concentration of 40 million cells/mL in 90% fetal bovine serum and 10% dimethylsulfoxide. Characterization of antigen profiles by multiparameter flow cytometry was performed on mononuclear cells prior to freezing. Antibody binding was evaluated on a FACSCalibur using the CellQuest software program (Becton-Dickinson, Mountainview, CA). The patient characteristics are summarized in **Table 1**.

### Genomic DNA extraction

Genomic DNA was extracted from 5–10×10^6^ cells following a standard phenol-chloroform protocol followed by an ethanol precipitation and resuspension in 10 mM Tris-HCl pH 8.0.

### Total RNA extraction

Total RNA was extracted from 20×10^6^ using an RNeasy Mini kit from Qiagen (Valencia, CA), eluting twice in 30 µL of RNAse-free water.

### Chromatin Immunoprecipitation Assay (ChIP)

10×10^6^ bone marrow cells were resuspended in 10 mL of DMEM and fixed with 1% formaldehyde at room temperature for 10 minutes. Reactions were quenched with 0.125 M glycine for 5 min, and the cells washed twice in cold PBS. Cells were lysed in 1.3 mL of lysis buffer (1% SDS+50 mM Tris-HCl pH 8.1+10 mM EDTA pH 8.0+protease inhibitors) on ice for 10 minutes and sonicated using a Branson microtip sonicator at 45% amplitude (Fisher scientific, Pittsburgh, PA) in order to achieve chromatin fragmentation to an average of 500 bp. Chromatin was pre-cleared with 100 µL of Protein A agarose beads (Roche, Indianapolis, IN) for 2 hours at 4°C, and then diluted 10-fold in dilution buffer (1.1% Triton X-100+0.01% SDS+167 mM NaCl+16.7 mM Tris-HCl pH 8.1+1.2 mM EDTA+protease inhibitors). One hundred microliters were set apart for the input sample. Immunoprecipitation (IP) reactions in chromatin from 1×10^6^ cells were carried overnight at 4°C with either 2.5 µg of anti-acetylated histone H3 lysine 9 antibody (Upstate, Catalog #: 07-352, lot: 31388) or an equal amount of IgG isotype control (Jackson ImmunoResearch). On the following day immune complexes were recovered by adding 25 µL of protein A agarose beads to each reaction, incubating at 4°C for 45 minutes and then spinning down at 3000 rpm. Each sample was washed 5 times, 10 minutes each time, as follows: 1× low salt wash (0.1% SDS+1% Triton X-100+2 mM EDTA+20 mM Tris-HCl pH 8.1+150 mM NaCl), 1× high-salt wash (0.1% SDS+1% Triton X-100+2 mM EDTA+20 mM Tris-HCl pH 8.1+500 mM NaCl), 1× LiCl wash (0.25 M LiCl+1% NP-40+1% deoxycholate+1 mM EDTA+10 mM Tris-HCl pH 8.1) and 2× TE pH 8.0 washes (10 mM Tris-HCl+1 mM EDTA). DNA from each of the IPs and the input sample was eluted twice for 20 minutes at room temperature in 100 µL of elution buffer (1% SDS+0.1 M NaHCO_3_) followed by an overnight incubation at 65°C to reverse the cross-linking. DNA samples were cleaned using a QIAquick PCR purification kit (QIAGEN, Valencia, CA) using the manufacturer's protocol, but using 30 µL of elution buffer for the final elution from the column. Enrichment was calculated by quantitative real-time PCR in a DNA Engine Opticon 2 real-time thermocycler from Biorad (Hercules, CA) using primers directed against the promoter regions of the *CD10*, *CD20*, *CD33* or *MPO* genes (for primer sequences see **Table S2**). Specific enrichment for H3K9ac was determined using the ΔΔC(t) method as previously described[29]. Samples were amplified using a ligation-mediated PCR protocol as previously described[30] and maintenance of enrichment was verified by quantitative PCR prior to submission for labeling and hybridization onto the NimbleGen human HG17 promoter tiling oligonucleotide microarray (2005-04-18_HGS17_min_promoter_set; design ID: 1871) containing 385,000 probes covering 24,134 promoters (1 kb and +500 bp around the transcription start-site, on average). ChIP-chip microarray data has been submitted to the GEO database for public access (accession number pending)

### DNA methylation analysis by HELP

The HELP assay was carried out as previously published[23] with slight modifications. One microgram of genomic DNA was digested overnight with either HpaII or MspI (NEB, Ipswich, MA). On the following day the reactions were extracted once with phenol-chloroform and resuspended in 11 µL of 10 mM Tris-HCl pH 8.0 and the digested DNA was used to set up an overnight ligation of the JHpaII adapter using T4 DNA ligase. The adapter-ligated DNA was used to carry out the PCR amplification of the HpaII and MspI-digested DNA as previously described[23]. Both amplified fractions were submitted to Roche NimbleGen, Inc. (Madison, WI) for labeling and hybridization onto a human HG17 custom-designed oligonucleotide array (50-mers) covering 25,626 HpaII amplifiable fragments (HAF) located at gene promoters and imprinted regions. HpaII amplifiable fragments are defined as genomic sequences contained between two flanking HpaII sites found within 200–2,000 bp from each other. Each HAF on the array is represent by 15 individual probes, randomly distributed across the microarray slide. HELP microarray data has been submitted to the GEO database for public access (accession number pending)

### Quantitative DNA methylation analysis by MassArray Epityping

Validation of HELP microarray findings was carried out by MALDI-TOF mass spectrometry using EpiTyper by MassArray (Sequenom, CA) on bisulfite-converted DNA as previously described[31]. MassArray primers were designed to cover the flanking HpaII sites for a given HAF, as well as any other HpaII sites found up to 2,000 bp upstream of the downstream site and up to 2,000 bp downstream of the upstream site, in order to cover all possible alternative sites of digestion (primer sequences available on **Table S2**).

### cDNA synthesis for Gene expression assays

Ten micrograms of total RNA were used to synthesize cDNA using Superscript III (Invitrogen, Carlsbad, California) using the manufacturer's protocol for qRT-PCR. Double-stranded cDNA synthesis for gene expression arrays was likewise carried out using Invitrogen's Superscript III, but a modified oligo-d(T) primer (5′GGCCAGTGAATTGTAATACGACTCACTATAGGGAGGCGGTTTTTTTTTTTTTTTTTTTTTTTT-3′) was used in lieu of the kit's oligo-d(T) primer. The second strand was synthesized for 2 hours at 16°C in a reaction containing 30 µL of 5×second strand buffer (Invitrogen), 90 µL of RNAse-free water, 3 µL dNTP 10 mM, 1 µL of BSA 1 µg/µL, 1 ul of E. coli DNA ligase 10 U/µL (Invitrogen), 4 µL E. coli DNA polymerase I 10 U/µL (Invitrogen) and 1 µL of RNase H 2 U/µL (Invitrogen). Finally, 2 µL of T4 DNA Polymerase 5 U/µL (Invitrogen) were added and the reaction incubated at 16°C for another 10 minutes. Reactions were cleaned with Qiagen's Qiaquick PCR purification kit and submitted for labeling and hybridization onto the NimbleGen standard human HG17 60mer 385,000 probe gene expression array (2005-04-20_Human_60mer_1in2; design ID: 1877). Gene expression microarray data has been submitted to the GEO database for public access (accession number pending)

### Quantitative real time PCR

The expression values of fifteen genes were validated by quantitative RT-PCR (qRT-PCR). cDNA was synthesized using the Superscript III First Strand kit from Invitrogen (Invitrogen's Superscript III) as per manufacturer's protocol. The ΔΔC(t) method was used to determine relative gene expression levels [32] using Power SYBRGreen from Applied Biosystems (Foster City, CA) and a DNA Engine Opticon 2 real-time thermocycler from Biorad (Hercules, CA). All primer sequences are available as supplementary data **(Table S2)**.

### Sample labeling and Microarray hybridizations

All samples for microarray hybridization were processed at the Roche NimbleGen Service Laboratory. Samples were labeled using Cy-labeled random primers (9mers) and then hybridized onto the corresponding microarray platform and scanned using a GenePix 4000B scanner (Axon Instruments) as previously described[33].

### Microarray quality control

All microarray hybridizations were subjected to extensive quality control using the following strategies. First, uniformity of hybridization was evaluated using a modified version of a previously published algorithm[34] adapted for the NimbleGen platform, and any hybridization with strong regional artifacts was repeated. Second, normalized signal intensities from each array were compared against a 20% trimmed mean of signal intensities across all arrays in that experiment, and any arrays displaying a significant intensity bias that could not be explained by the biology of the sample were excluded. Finally, replicate reproducibility was estimated by using scatter plots and a Pearson correlation matrix, and any clear outliers were excluded from further analysis **(Figures S1A, S1B and S1C).**

### Acetyl histone H3 lysine 9 ChIP-chip analysis

The H3K9 acetylation status at each promoter was computed by taking the log-ratio between the probe intensities of the ChIP product and input chromatin, which were co-hybridized on the same assay. For each of the 24,134 promoter regions, we computed the moving average of the log-ratio along its 15 probes with a window size of 3 probes, and found the maximum value of the moving averages of over each region covered by 15 probes. The maximum values served as the summary of histone H3 lysine 9 acetylation of the promoter regions and were used to correlate with gene expression profiles. All chips that passed primary quality control showed a bimodal distribution with a distinct lower mode. We took these lower modes to indicate non-enriched fragments, and normalized across arrays by lining up these modes.

### HELP data processing and analysis

Signal intensities at each HpaII amplifiable fragment were calculated as a robust (25% trimmed) mean of their component probe-level signal intensities. Any fragments found within the level of background MspI signal intensity, measured as 2.5 mean-absolute-differences (MAD) above the median of random probe signals, were categorized as “failed.” These “failed” loci therefore represent the population of fragments that did not amplify by PCR, whatever the biological (e.g. genomic deletions and other sequence errors) or experimental cause. On the other hand, “Methylated” loci were so designated when the level of HpaII signal intensity was similarly indistinguishable from background. PCR-amplifying fragments (those not flagged as either “methylated” or “failed”) were normalized using an intra-array quantile approach wherein HpaII/MspI ratios are aligned across density-dependent sliding windows of fragment size-sorted data. Analysis of normalized data revealed the presence of a bimodal distribution. For each sample a cutoff was selected at the point that more clearly separated these two populations and the data were centered around this point. Each fragment was then categorized as either methylated, if the centered log HpaII/MspI ratio was less than zero, or hypomethylated if on the other hand the log ratio was greater than zero.

### Gene expression data processing and analysis

Raw data (.pair files) from the scanner were processed with Roche NimbleGen's version of the RMA algorithm (without background correction) in NimbleScan 2.3 software. Gene expression microarray assays were performed in quadruplicate for each patient and 16 out of the 20 chips were selected on the basis of standard quality metrics and uniformity of hybridization. Next we selected 10 chips with the least evidence of technical artifact compared with the median across all chips, and constructed expression profiles for each of the five samples by averaging each pair of replicate chips among these 10.

### Descriptive statistics

Noise in gene expression and other microarray measures was estimated as the standard error of the averages of the measures across technical replicates. For the ChIP-chip arrays the average standard error was 0.58. Gene expression values were processed by NimbleScan 2.3 software using a quantile normalization, which depresses estimates of variance at either end of the intensity distribution relative to the middle. Most of our measures of interest came from the middle range of intensities and their average estimated standard error was 0.30. For HELP, the average standard error of the quantile-normalized ratio estimates was 0.21. For between-measures correlation analysis we selected genes whose measured standard deviation was at least 2.5 times the standard error of the averages of replicates. Signal to noise ratio (SNR) for all the platforms was defined as the ratio of variation across samples compared to the variation between replicates of the same sample

### Clustering analysis

Unsupervised clustering of HELP and ChIP-chip data using hierarchical clustering (correlation distance and complete linkage) [35], correspondence analysis [36] and principal component analysis [37] were performed using the open-source statistical software R [38] and the BioConductor package MADE4 [39]. Since there are 10 distinct ways in which 5 samples can be divided into two groups of 2 and 3 samples each, it was calculated that there was a probability of 1 in 10 that this analysis would achieve accurate separation of samples by lineage due to chance.

Supervised analysis was done using a standard T-test with leukemic lineage (AML or ALL) as the dichotomous variable. A significance level of p<0.02 was chosen for all three platforms. In the case of the methylation data, a second criterion of a difference>1.5 between the mean of the two populations was added in order to increase the likelihood of detection of biologically significant changes in methylation levels.

### Pathway analysis

Using the Ingenuity Pathway Analysis software (IPA) (Redwood City, CA) we carried out an analysis of the biological information retrieved by each of the individual platforms alone, and compared it to the information obtained by the integrated analysis of all three platforms. Enrichment of genes associated with specific canonical pathways was determined relative to the Ingenuity knowledge database for each of the individual platforms and the integrated analysis at a significance level of p<0.01. Biological networks captured by the different microarray platforms were generated using IPA and scored based on the relationship between the total number of genes in the specific network and the total number of genes identified by the microarray analysis.

## Supporting Information

Text S1 Supplementary methods: Model for testing likelihood of correlations between measures
(0.06 MB DOC)

Figure S1 Correlation matrix between replicate arrays and different biological samples for each platform. Panel A: Correlation matrix for all normalized HELP microarrays that passed hybridization quality control. Panel B: Correlation matrix for H3K9-acetyl ChIP-chip arrays that passed initial quality control. Panel C: Correlation matrix for the ten gene expression arrays selected for analysis after quality control assessment of all hybridizations.
(3.52 MB TIF)

Figure S2 Technical validations by single locus studies for each microarray platform. Panel A: Correlation between qRT-PCR findings (x axis: Delta C(t) to GAPDH) and gene expression arrays (y axis: log2 intensity). The overall negative correlation (r = −0.72) confirms the good quality of the gene expression array data. Panel B: Correlation between H3K9-acetyl ChIP-chip (x axis: log H3K9ac/Input) and fold enrichment over non-specific IgG by qChIP (y axis). Since H3K9ac ChIP-chip data display a bimodal distribution, the lowest point between the two peaks (log H3K9acetyl = 2.5) was selected as a cutoff for comparison with qChIP. While most points below this cutoff point show minimal enrichment by qChIP, points to the right of this cutoff clearly show increasing amounts of enrichment with increasing log H3k9ac/input values. Panel C: Relationship between DNA methylation by HELP (x axis: log HpaII/MspI) and percent cytosine methylation measured by MALDI-TOF mass spectrometry.
(0.86 MB TIF)

Figure S3 Unsupervised clustering using of epigenomic data principal component analysis (PCA). Unsupervised clustering of DNA methylation and H3K9 acetylation data using PCA separated the leukemia samples according to lineage along the first principal component. Panel A: Two-dimensional representation of PCA of DNA methylation data. ALL samples (in red) readily cluster apart from AML samples (in blue) along the first principal component (x axis) (left); and heatmap of top 100 genes from the first principal component (right). Genes are shown on the rows and samples on the columns, and data were row-centered. Low values represented in blue and high values in red. Panel B: Two-dimensional representation of PCA of H3K9-acetyl ChIP-chip data. ALL samples (in red) and AML samples (in blue) readily segregated along the first principal component (x axis) (left); and heatmap of top 100 genes from the first principal component (right). Genes are shown on the rows and samples on the columns, and data were row-centered. Low values represented in blue and high values in red.
(1.57 MB TIF)

Figure S4 Highest scoring networks from Ingenuity Pathway Analysis comparing the individual platforms and the integrated analysis. Biological gene networks identified as differentially regulated in ALL versus AML. Networks were generated using the genes identified through the analysis of the individual platforms or from the genes identified when information from all three platforms was integrated. Panel A: The top two scoring networks identified using H3K9 acetylation data centered around HoxA9 and APP. Genes that were present in the analysis gene list appear colored in grey while genes identified indirectly appear in white. Panel B: The top two scoring networks identified using DNA methylation data centered around TNF and NFkB. Panel C: The top two scoring networks identified using gene expression data centered around TERT and NFkB. Panel D: The top two scoring networks identified using data from the integration of all three platforms centered around TNF and TP53. Panel E: The biological network centering around MYC was among the highest scoring networks (network #6) when using the integrated data, while none of the individual platforms succeeded in identifying the MYC network directly.
(2.90 MB TIF)

Table S1 Canonical pathway analysis
(0.03 MB XLS)

Table S2 Primer sequences
(0.05 MB XLS)
