# Supplementary material for: An Integrative Genomic and Epigenomic Approach for the Study of Transcriptional Regulation
Source: PLoS One. 2008 Mar 26;3(3):e1882. doi: 10.1371/journal.pone.0001882 (PMC2266992; doi:10.1371/journal.pone.0001882)
Supplement: Text S1 — Supplementary methods: Model for testing likelihood of correlations between measures (0.06 MB DOC) [file pone.0001882.s001.doc]

**Supplementary methods:**

**Model for testing likelihood of correlations between measures**

The focus of this paper is the relationship of measures between platforms. Data was available for all three modalities (expression, histone acetylation, and methylation) on 17,180 genes. We show that the strong relationships we see among measures are not likely to occur by chance, even though our sample size (five) is small.

In our study of correlation we selected genes of high variance for our analysis so that true correlations would be better measured. We found a large number of very high correlations between platforms. If all our genes varied independently then the number of high correlations that we observed would be so large that such an outcome would be astronomically small (the probability of obtaining a mean of 0.469 from 393 independent correlations of unrelated data is p < 10^-53^). However it might be objected that variation in gene expression or epigenetic regulation does not occur independently. Thus a possible problem with our analysis would appear if the case were that most genes are correlated (so that only one or two principal components could account for most of the variation). Under this scenario a large number of high correlations could occur when comparing two measures from five individual patient samples. However, we demonstrate below that this cannot be an explanation of our large number of high correlations, because under these circumstances the permutation distribution of correlation would also frequently achieve high numbers of high correlations some of the time.

If the situation proposed above were true, then we should not see as many high correlations in a set of genes selected so that their measures are as much statistically independent as possible. We could test this for example in our acetylation and expression data sets by selecting genes with expression measures as uncorrelated as possible to a given starting set of genes. 65 such high variance genes in both expression and acetylation were selected to be as independent as possible in each platform separately. When we permuted the sample labels and re-calculated the correlations, we found that none of the 119 non-identical permutations of sample labels gave a distribution of correlations whose high percentiles exceeded those of the actual ordering (80^th^ percentile of observed was 0.822; maximum 80^th^ percentile of all 119 non-identity permutations was 0.805). We conclude that the levels of association that we observe in this study are unlikely to be achieved by any random assortment of five samples. Therefore random effects that could happen in a small sample of highly correlated data are exceptionally unlikely to account for our observation, and would not be a viable alternative explanation to systemic high correlations between acetylation and expression or any of the other measures in our analysis. Thus, it is extremely unlikely that adding more samples would substantially reduce the number of high correlations that we observe.

**R CODE FOR SELECTING GENES AND TESTING PERMUTATIONS**

# select genes whose expr & acet measures are fairly independent, to get maximal statistical power and make it difficult to achieve high correlations by chance

# variables: acet – acetylation measures; expr – expression measures; acet.sd – SD of genes in acet ; acet.to.expr – corresponding indices from acetylation to expression

nn <- which( acet.sd > quantile( acet.sd, .95 ) & expr.sd[ acet.to.expr ] > quantile( expr.sd, .9) )

mm <- nn[1:6]

pp.a <- prcomp( t( acet[ mm, ] ) )

pp.e <- prcomp( t( expr[ acet.to.expr[ mm] , ] ) )

for ( ii in 6:length(nn)) {

  pp.acet <- prcomp( t( acet[ c( mm, nn[ii] ), ] ) )

  pp.expr <- prcomp( t( expr[ acet.to.expr[ c( mm, nn[ii] )] , ] ) )

  if ( pp.acet$sdev[4] / pp.acet$sdev[1] > .95 * pp.a$sdev[4] / pp.a$sdev[1]   &

       pp.expr$sdev[4] / pp.expr$sdev[1] > pp.e$sdev[4] / pp.e$sdev[1] ) {

    mm <- c( mm, nn[ii] )

    pp.a <- pp.acet

    pp.e <- pp.expr

  }

}

# plot and record percentiles of permutation distribution of correlations

# NB. limit.density is a variant of density() for a closed interval

plot( limit.density( cors.acet.expr[mm],-1,1))

cc <- numeric()

vv <- matrix(nr=1000,nc=4)

for ( ii in 1:1000 ) {

  kk <- sample(5)

  if ( all( kk==1:5) ) next

  for ( jj in 1:length(mm))

    cc[jj] <- cor( acet[mm[jj],], expr[acet.to.expr[mm[jj]], kk ] )

  lines( limit.density( cc, -1, 1), col=4, lty=2)

  vv[ii,]<- quantile( cc, 5:8/10)

}
