# Supplementary figures and images for: An Integrative Genomic and Epigenomic Approach for the Study of Transcriptional Regulation
Source: PLoS One. 2008 Mar 26;3(3):e1882. doi: 10.1371/journal.pone.0001882 (PMC2266992; doi:10.1371/journal.pone.0001882)

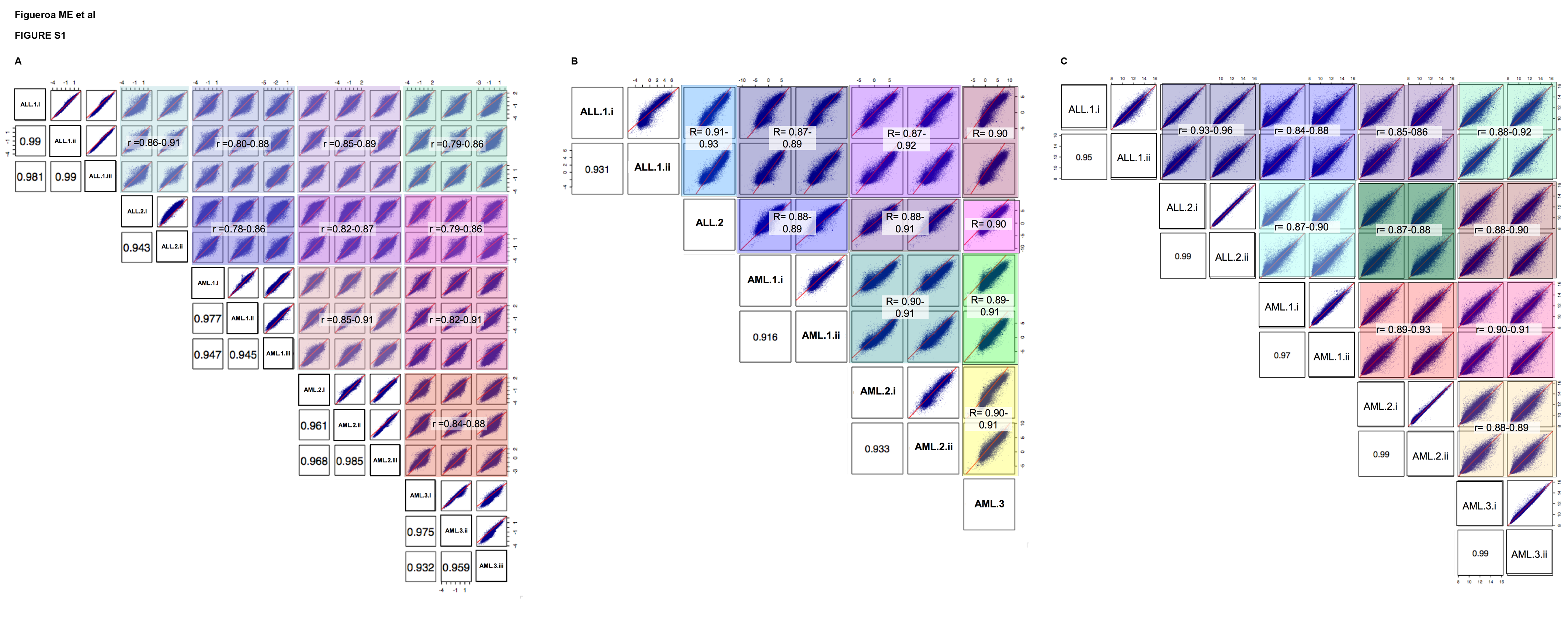

Supplement: Figure S1 — Correlation matrix between replicate arrays and different biological samples for each platform. Panel A: Correlation matrix for all normalized HELP microarrays that passed hybridization quality control. Panel B: Correlation matrix for H3K9-acetyl ChIP-chip arrays that passed initial quality control. Panel C: Correlation matrix for the ten gene expression arrays selected for analysis after quality control assessment of all hybridizations. (3.52 MB TIF) [file pone.0001882.s002.tif]

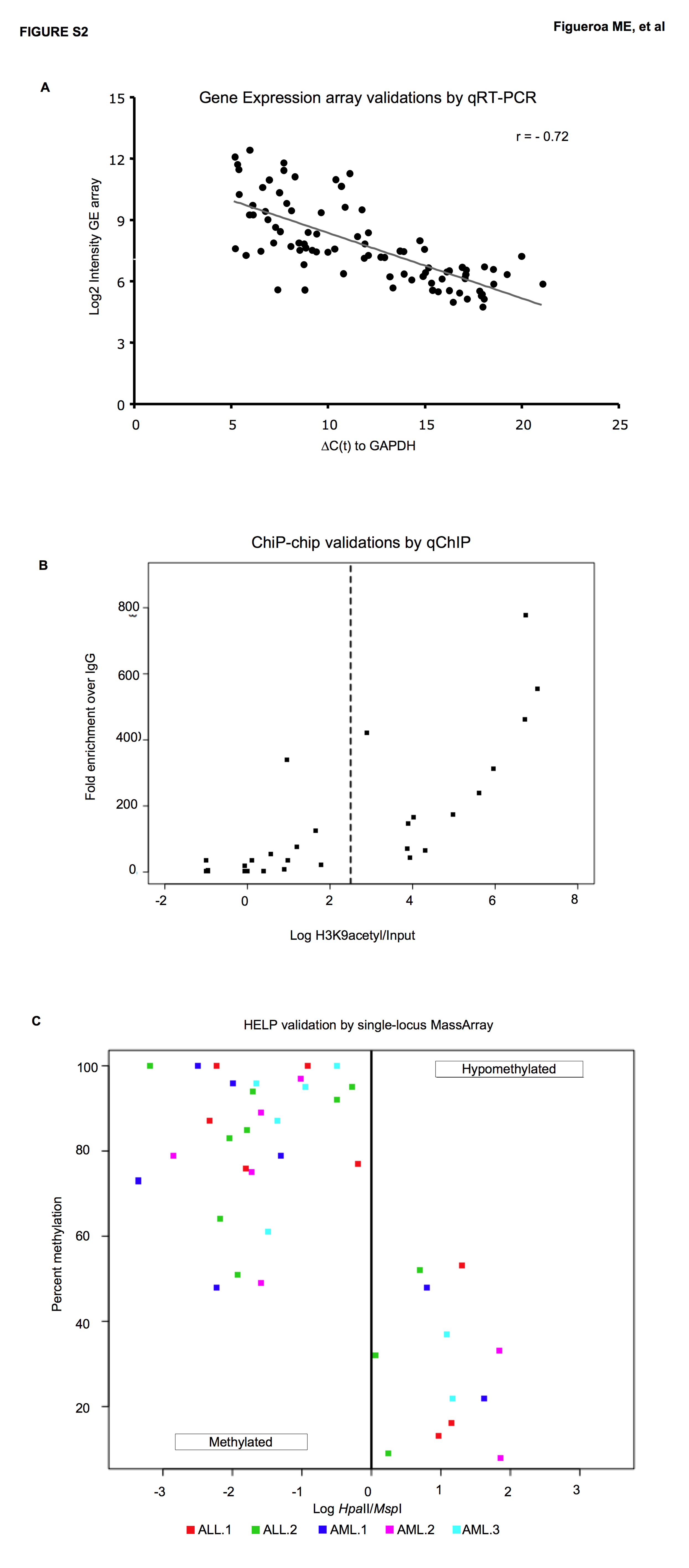

Supplement: Figure S2 — Technical validations by single locus studies for each microarray platform. Panel A: Correlation between qRT-PCR findings (x axis: Delta C(t) to GAPDH) and gene expression arrays (y axis: log2 intensity). The overall negative correlation (r = −0.72) confirms the good quality of the gene expression array data. Panel B: Correlation between H3K9-acetyl ChIP-chip (x axis: log H3K9ac/Input) and fold enrichment over non-specific IgG by qChIP (y axis). Since H3K9ac ChIP-chip data display a bimodal distribution, the lowest point between the two peaks (log H3K9acetyl = 2.5) was selected as a cutoff for comparison with qChIP. While most points below this cutoff point show minimal enrichment by qChIP, points to the right of this cutoff clearly show increasing amounts of enrichment with increasing log H3k9ac/input values. Panel C: Relationship between DNA methylation by HELP (x axis: log HpaII/MspI) and percent cytosine methylation measured by MALDI-TOF mass spectrometry. (0.86 MB TIF) [file pone.0001882.s003.tif]

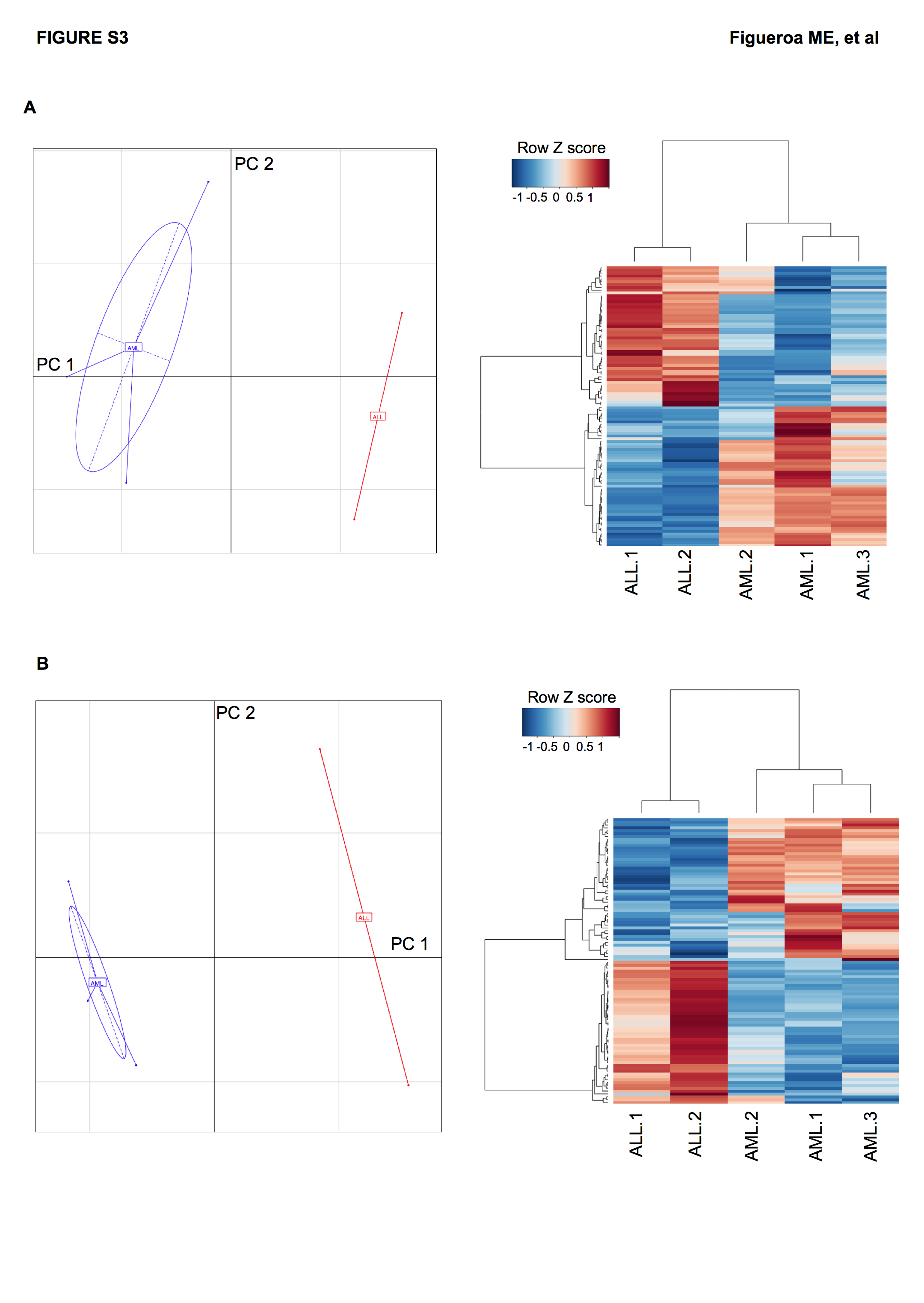

Supplement: Figure S3 — Unsupervised clustering using of epigenomic data principal component analysis (PCA). Unsupervised clustering of DNA methylation and H3K9 acetylation data using PCA separated the leukemia samples according to lineage along the first principal component. Panel A: Two-dimensional representation of PCA of DNA methylation data. ALL samples (in red) readily cluster apart from AML samples (in blue) along the first principal component (x axis) (left); and heatmap of top 100 genes from the first principal component (right). Genes are shown on the rows and samples on the columns, and data were row-centered. Low values represented in blue and high values in red. Panel B: Two-dimensional representation of PCA of H3K9-acetyl ChIP-chip data. ALL samples (in red) and AML samples (in blue) readily segregated along the first principal component (x axis) (left); and heatmap of top 100 genes from the first principal component (right). Genes are shown on the rows and samples on the columns, and data were row-centered. Low values represented in blue and high values in red. (1.57 MB TIF) [file pone.0001882.s004.tif]

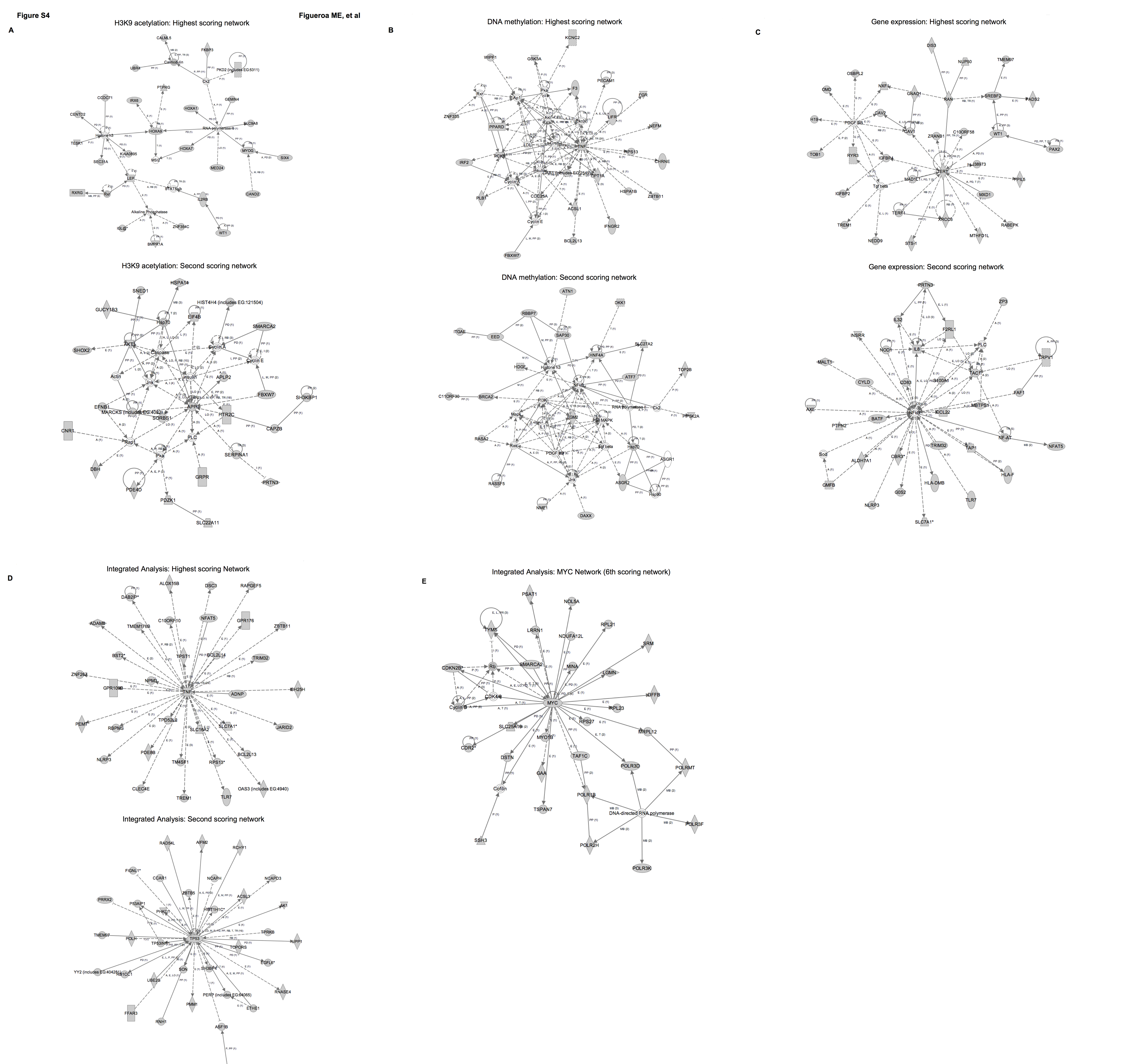

Supplement: Figure S4 — Highest scoring networks from Ingenuity Pathway Analysis comparing the individual platforms and the integrated analysis. Biological gene networks identified as differentially regulated in ALL versus AML. Networks were generated using the genes identified through the analysis of the individual platforms or from the genes identified when information from all three platforms was integrated. Panel A: The top two scoring networks identified using H3K9 acetylation data centered around HoxA9 and APP. Genes that were present in the analysis gene list appear colored in grey while genes identified indirectly appear in white. Panel B: The top two scoring networks identified using DNA methylation data centered around TNF and NFkB. Panel C: The top two scoring networks identified using gene expression data centered around TERT and NFkB. Panel D: The top two scoring networks identified using data from the integration of all three platforms centered around TNF and TP53. Panel E: The biological network centering around MYC was among the highest scoring networks (network #6) when using the integrated data, while none of the individual platforms succeeded in identifying the MYC network directly. (2.90 MB TIF) [file pone.0001882.s005.tif]
